# Facile Fabrication of Superhydrophobic Surfaces with Hierarchical Structures via Water Vapor Condensation

**DOI:** 10.1002/smtd.202502305

**Published:** 2026-03-01

**Authors:** Jeonghan Kang, Seung Yoon Nam, Sungho Lee

**Affiliations:** ^1^ Department of Mechanical Engineering Dong‐A University Busan Republic of Korea

**Keywords:** hierarchical structure, self‐cleaning, superhydrophobic surface, water vapor condensation, wettability

## Abstract

Surfaces with a water contact angle greater than 150° are defined as superhydrophobic surfaces, exhibiting characteristics such as water repellency, self‐cleaning ability, and extremely low friction with water droplets. Most superhydrophobic surfaces possess micro‐ or nanoscale hierarchical structures; however, the fabrication of superhydrophobic surfaces typically requires considerable time and cost. Herein, we report a facile method for fabricating hierarchical structures via condensation of water vapor to address these problems. The size of the hierarchical structures can be controlled by adjusting the condensation time. The hierarchical structures via water vapor condensation exhibit the features of superhydrophobic surfaces, as confirmed by measurements of contact angle, sliding angle, and droplet impact behavior. As a feasible application, a self‐cleaning test is also carried out. The facile fabrication method in this study is expected to be easily applicable for hierarchical structure formation and further extended to large‐area sample production.

## Graphical abstract

In this work, we demonstrate a facile and robust strategy for fabricating superhydrophobic surfaces by introducing micro‐scale hierarchical structures via controlled water vapor condensation. The modulation of condensation time enables the fabrication of dual‐level hierarchical structures, which were systematically validated through contact angle and sliding angle measurements to confirm their superior non‐wetting performance and structural stability.

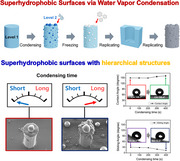

## 1 Introduction

Superhydrophobic surfaces refer to surfaces where the contact angle (CA) with water droplets exceeds 150 degrees. A representative example of such surfaces is the “lotus leaf effect.” Lotus leaves exhibit water‐repellent and self‐cleaning properties, which help keep their surfaces clean. The surface of a lotus leaf has a hierarchical structure, where nanostructures are formed on top of micro‐protrusions. Due to this hierarchical structure, water droplets can easily roll off the surface, effectively removing pollutants as they move [1, 2, 3]. Research on the fabrication of lotus‐leaf‐inspired superhydrophobic surfaces has been actively conducted [4, 5, 6, 7, 8, 9]. To fabricate a superhydrophobic surface, it is essential to maintain an air layer between the microstructures, forming the Cassie‐Baxter state, in which water droplets rest on top of the structures [10]. The difference between the internal and external pressures of the droplets can be described using Laplace pressure. When the Laplace pressure becomes negative, the droplets collapse into the microstructures, transitioning from the Cassie‐Baxter state to the Wenzel state. Therefore, superhydrophobic surfaces should be designed to maintain a positive Laplace pressure, and various studies have proposed specific microstructure design methods to achieve this condition [11, 12, 13]. Although superhydrophobic surfaces can be realized with microstructures alone, many designs incorporate hierarchical structures to maximize the superhydrophobic effect. Hierarchical structures not only reduce the pinning effect of water droplets but also increase the Laplace pressure, thereby enhancing fluid repellency [14, 15, 16, 17, 18, 19, 20, 21]. Despite advances in micro/nano fabrication technologies that have enabled the creation of diverse hierarchical structures, most fabrication methods still require expensive equipment and complex processes, resulting in high production costs.

Herein, we introduce a facile and cost‐effective fabrication strategy for a superhydrophobic surface by forming hierarchical structures through the condensation of water vapor on a pre‐fabricated microstructure. The process involves cooling the pre‐fabricated microstructure using a thermoelectric element to condense water vapor from the air, followed by freezing the condensed water droplets by further lowering the temperature. The resulting hierarchical structure was uniformly observed throughout the surface—from the top to the bottom of the microstructures. Moreover, the size of the hierarchical features could be controlled by adjusting the condensation time. To verify the performance of the superhydrophobic surface, contact angle and sliding angle measurements were carried out, yielding values of 160.4° and 13.3°, respectively. As a feasible application, a self‐cleaning test was also conducted. These results confirmed the excellent superhydrophobic characteristics of the fabricated surfaces. This fabrication strategy offers the advantage of easily forming hierarchical structures regardless of the underlying microstructure's shape, enabling the low‐cost production of superhydrophobic surfaces. We believe this method has strong potential for application in research and various industries related to superhydrophobic surface development.

## 2 Experimental Section

### 2.1 Fabrication of Microstructures

The master mold with a micropillar array having a diameter of 40 µm, a height of 80 µm, and an aspect ratio (AR) of 2 was fabricated in our previous study [13]. For the control sample, the micropillar array was replicated from the master mold using polyurethane acrylate (PUA) (311RM, Minuta, Korea). An adequate amount of PUA was spread on the surface and cured for 15 s under UV irradiation at a power of 8 mW.

### 2.2 Fabrication of Hierarchical Structures

To form hierarchical structures via water vapor condensation on the microstructures, the sample was placed on a thermoelectric cooler (TEC) under conditions of 19°C and 54% relative humidity. After a certain condensation time, the condensed water droplets on the microstructures were frozen by cooling the TEC to −30°C. The hierarchical structures, consisting of frozen microscale droplets on the micropillars, were obtained. This hierarchical structure was then replicated using polydimethylsiloxane (PDMS) to fabricate a negative‐tone hierarchical mold, and a subsequent replica was fabricated using PUA from the PDMS mold. Thus, a superhydrophobic surface with hierarchical structures was prepared.

### 2.3 Morphological Characterization of Hierarchical Structures

The morphology of the hierarchical structures was examined using an optical microscope (LV150L, Nikon, Japan) and a scanning electron microscope (SEM) (S‐4800, Hitachi, Japan). Prior to SEM observation, a thin Pt layer was sputtered onto the surface of the hierarchical structures to prevent electron charging.

### 2.4 Surface Treatment

To induce hydrophobicity, the surface of the hierarchical structures was treated with silane (L‐SAM, Minuta, Korea). L‐SAM was sprayed onto the surface and heated on a hot plate at 90°C for 1 h. Before silane treatment, the sample was exposed to a plasma cleaner (PS‐100, Plasol, Korea) with a power of 100 W for 120 s to enhance chemical bonding between the hierarchical structures and the silane monolayer.

### 2.5 Wettability Test

The contact angle (CA) and sliding angle (SA) of the hierarchical structures were measured using a contact angle analyzer. For CA measurement, a 5 µL water droplet was dropped onto the surface, and the droplet shape was analyzed. For SA measurement, a 10 µL water droplet was used, and the tiltable stage was tilted at a precise rate of 1° s^−^^1^. The experimental results were averaged over at least five repetitions. Finally, the bouncing behavior of water droplets on the sample was recorded at 1000 fps using a high‐speed camera (XSM‐4K Veloce, IDT Cameras, USA).

## 3 Result and Discussion

### 3.1 Fabrication and Design

**FIGURE 1:**
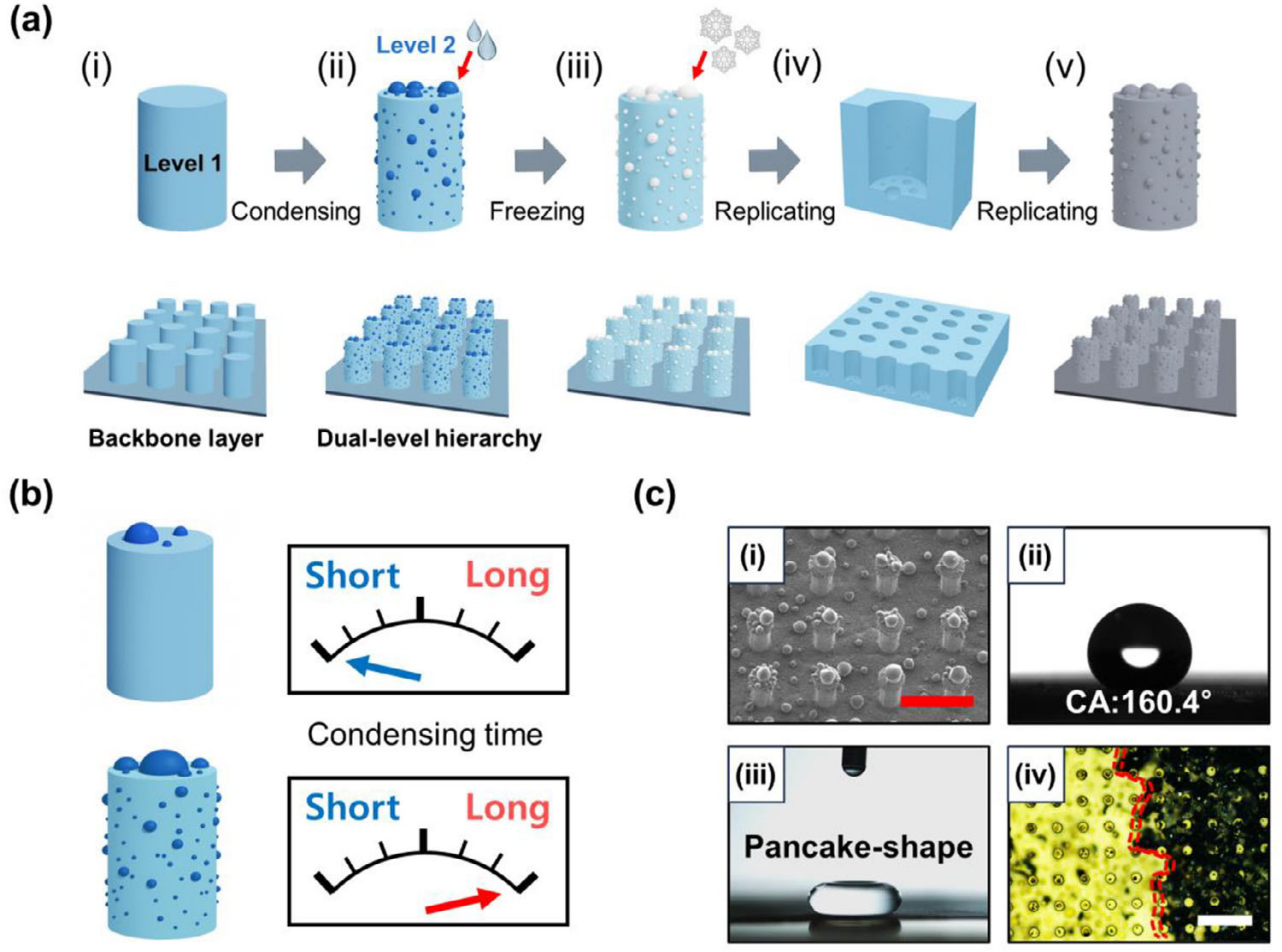
Overall schematic of this study. (a) Illustration of the fabrication process of the hierarchical structures via water vapor condensation: (i) micropillar array (backbone layer), (ii) condensation of water droplets on the surface, (iii) formation of hierarchical structures by freezing the condensed water, (iv) replication of the hierarchical structures (negative tone), and (v) replication of the hierarchical structures (positive tone). (b) Schematics of morphology controllability depending on the condensation time. (c) Morphology and superhydrophobic performance of the hierarchical structures: (i) SEM image, (ii) contact angle measurement, (iii) water droplet bouncing behavior with a pancake shape, and (iv) self‐cleaning test. Scale bar: 200 µm.

This section describes the fabrication and experimental results of the dual‐level hierarchical structures. Specifically, Level 1 serves as the micro‐pillar backbone, while Level 2 is established by the condensed water droplets on these pillar surfaces. To fabricate these structures, the secondary hierarchical features (ranging from sub‐micro to microscale) were formed by utilizing the condensation of water vapor onto the existing backbone layer (micro‐scale pillars). This fabrication method can be easily applied to various types of microstructures, including those with complex geometries or large‐area substrates. The mechanism of this fabrication method is illustrated in detail in Figure 1.

As shown in Figure 1a‐i, a sample consisting of micropillar structures with a diameter of 40 µm, a height of 80 µm, and an aspect ratio (AR) of 2 was prepared over an area of 30 mm × 30 mm as a backbone layer (Figure S1). To induce condensation of water vapor on the microstructured surface, the sample was placed on a thermoelectric cooler (TEC) and cooled (Figure 1a‐ii). After a certain condensation time, microscale droplets were formed on the surface of the micropillars (Figure 1a‐ii). By cooling the TEC to −30°C, the condensed droplets were frozen in place on the microstructures (Figure 1a‐iii). Through the processes illustrated in Figure 1a‐ii and iii, hierarchical structures consisting of frozen microscale droplets on the micropillars were obtained. This hierarchical structure was replicated using polydimethylsiloxane (PDMS) to fabricate a negative‐tone hierarchical mold (Figure 1a‐iv).

An important reason for employing PDMS in the replication of hierarchical structures lies in its immiscibility with water. While water is well known as a representative polar substance, PDMS is regarded as a nonpolar material [22]. This contrast implies that water and PDMS do not readily mix, which can be quantitatively described using the solubility parameter (δ) [23, 24]:

$$\delta ={\left(\frac{U}{V}\right)}^{0.5}$$

where, *U* and *V* are the molar internal energy (cal mol^−1^) and the molar volume (cm^3^ mol^−1^), respectively. According to the literature, the δ value of PDMS was reported to be 7.3 cal^1/2^ ∙cm^−3/2^, whereas that of water is 23.4 cal^1/2^ ∙cm^−3/2^. In a ranking of 38 solvents in terms of miscibility with PDMS, water was placed last (38th out of 38), clearly indicating its poor compatibility [24]. Therefore, in the replication of hierarchical structures fabricated via water condensation, the use of PDMS, which does not mix with water, is an important characteristic for this fabrication method.

Finally, UV imprint lithography using polyurethane acrylate (PUA) was employed to complete the fabrication of hierarchical microstructures via water vapor condensation [25, 26]. As shown in Figure 1, this fabrication method is highly facile and cost‐effective. Moreover, the size of the condensed droplets can be controlled by adjusting the condensation time, enabling the fabrication of various types of hierarchical structures from a single micropillar template (Figure 1b). As with most micro‐hierarchical structures, the fabricated hierarchical structures exhibited superhydrophobic behavior. As shown in Figure 1c, the performance of the superhydrophobic surfaces, such as contact angle with a value of 160.4°, pancake‐like droplet shape, and self‐cleaning properties were successfully demonstrated.

### 3.2 Morphology

**FIGURE 2:**
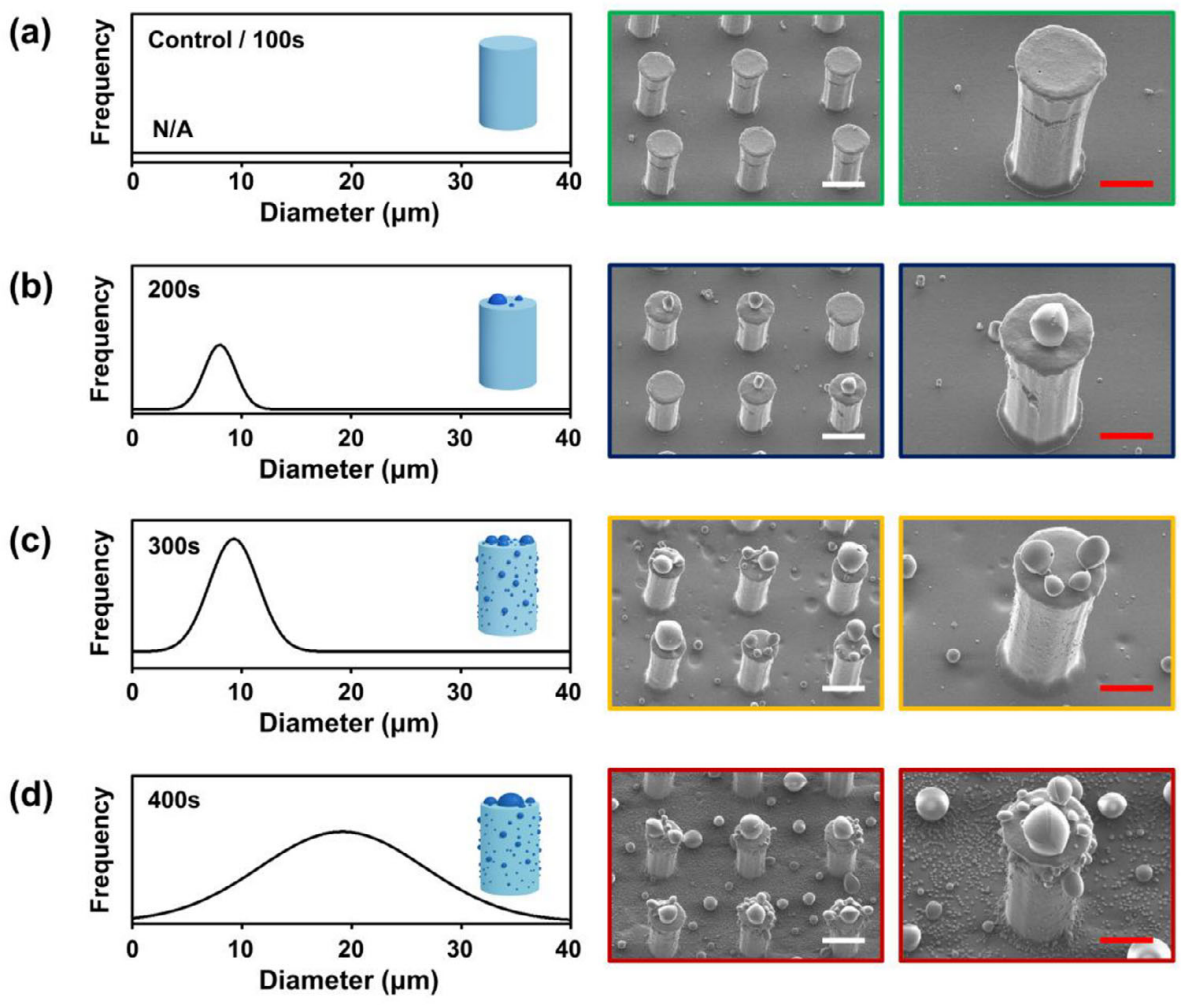
Fabrication results of hierarchical structures via water condensation. As the condensation time increases, both the size and the number of condensed droplets increase. Condensation times: (a) 100 s, (b) 200 s, (c) 300 s, and (d) 400 s. Scale bars: 50 µm (white) and 25 µm (red).

As mentioned in Section 3.1, the morphology of hierarchical structures is different depending on the condensation time, and Figure 2 shows the morphology change of hierarchical structures fabricated under different condensation times. The droplet size on the micropillars was controlled by adjusting the condensation time, and the resulting hierarchical morphologies are shown in Figure 2 and Figures S2 and S3. Condensation was carried out for 100 s, 200 s, 300 s, and 400 s under 19°C and 54% relative humidity conditions. For a condensation time of 100 s, water vapor condensation was not observed, suggesting that the condensation time of 100 s was insufficient to form the hierarchical structures (Figure 2a). In contrast, droplets with average diameters of 11.2 µm, 13.7 µm, and 19.5 µm were formed after 200 s, 300 s, and 400 s, respectively (Figure 2b–d). With increasing condensation time, both the droplet density and average droplet size were increased. This trend can be quantitatively analyzed using Gaussian distribution fitting, and the Gaussian distributions in Figure 2 confirm that both the average droplet size and the density of condensed droplets increased with longer condensation time. Specifically, droplet densities (droplet diameter > 5 µm) of 35, 139, and 208 droplets per 0.3 mm^2^ were observed at 200 s, 300 s, and 400 s, respectively, demonstrating that condensation time can modify the morphology of the hierarchical structures (Table 1).

**TABLE 1:** Quantitative analysis of condensation droplet characteristics with respect to the condensing time, including average droplet diameter (>5 µm), number density, and surface area coverage.

Condensing time (s)	Diameter (>5 µm)	Density (n/mm^2^)	Area coverage (%)
200	11.2	117	22
300	13.7	463	48
400	19.5	693	72

Two important factors to be considered during the fabrication of hierarchical structures through water vapor condensation are vibration control during the droplet formation on the TEC and surface properties of microstructures.

**FIGURE 3:**
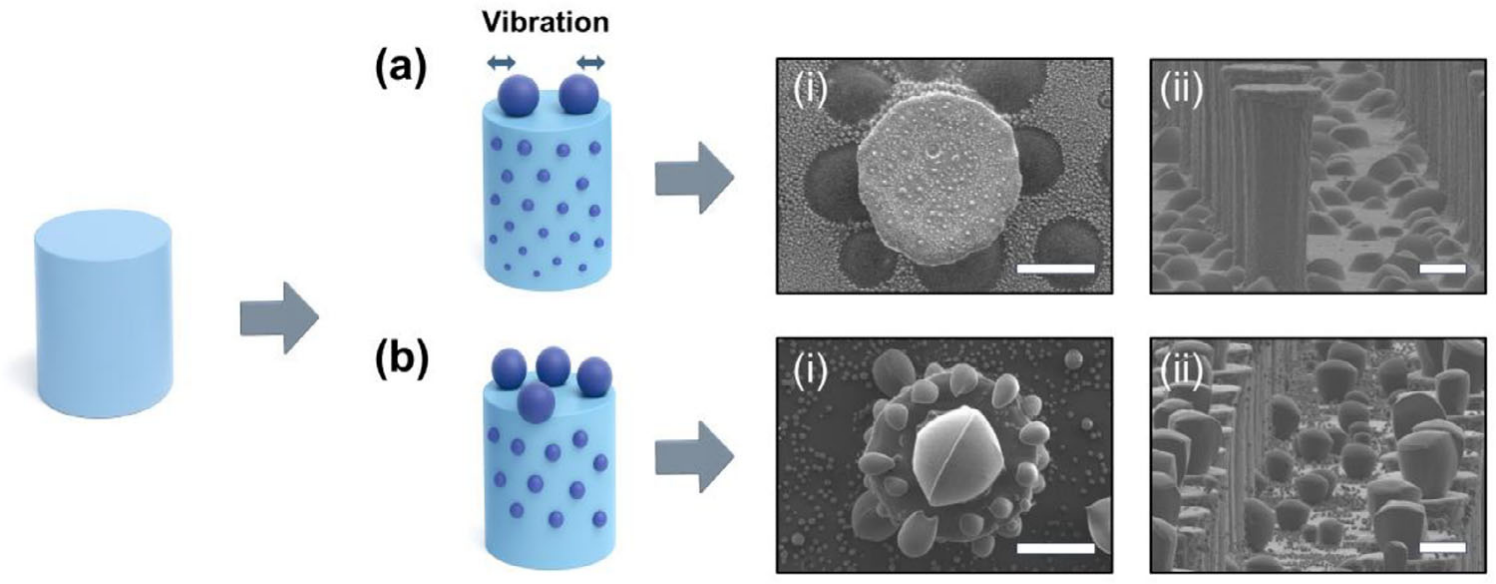
Hierarchical structures fabricated via water condensation with and without vibration control (condensation time: 400 s). When vibration was not controlled, condensed droplets on the microstructures were observed to be displaced around the structures, even for the same condensation duration. (a) without vibration control, (b) with vibration control. Scale bars: 20 µm.

The first one is the vibration control. When droplet condensation occurs on micropillars without vibration control, microscale droplets easily detach and fall near the micropillars due to external vibrations, hindering the formation of hierarchical structures (Figure 3a). Figure 3 compares condensation results after 400 s with and without vibration control. In Figure 3a, hierarchical structures formed only on the micropillar sidewalls, while a few small droplets remained on the top surfaces due to droplet fall. As shown in Figure 3a, the condensed droplet fell from the top surface of the micropillar and accumulated around its base. In contrast, when vibration was controlled (Figure 3b), droplets were stably formed on the tops of the micropillars, leading to the formation of hierarchical structures.

The other one is the surface properties of microstructures. During the formation of hierarchical structures, the resulting morphology is different depending on whether the surface of microstructures is hydrophilic or hydrophobic (Figure S5). To investigate how surface wettability affects the formation of hierarchical structures, the experiment with the condensation time of 400 s was conducted on two types of micropillar surfaces: one made of untreated PUA (hydrophilic with a CA of 78.5°) and another made of hydrophobically surface‐treated PUA (contact angle of 106.1°). Despite having the same condensation time, the hydrophobically treated PUA surface showed almost no formation of hierarchical structures; instead, droplets tended to detach and roll off the side of the micropillars. In contrast, well‐defined hierarchical structures were successfully formed on the untreated PUA surface without the roll off of droplets. This result can be attributed to the lower friction between water droplets and hydrophobic surfaces, causing the droplets to detach easily from the micropillars. The work of adhesion (*W*) of the water droplet to the solid phase can be expressed by the following equation:

$$W-\pi_{e}=\gamma \left(1+\cos\theta \right)$$

where, π_*e*_ and γ are the reduction in surface energy of the solid due to absorption and the surface energy of water (72 mN m^−1^), respectively, and θ is the contact angle [27, 28, 29, 30, 31]. Assuming that π_*e*_ is zero, the work of adhesion of the untreated PUA surface (hydrophilic) was calculated to be 86.5 mN m^−1^, while that of the surface‐treated PUA (hydrophobic) was 51.8 mN m^−1^. The result indicates that the work of adhesion on the hydrophobic surface was approximately 40% lower, which implies that water droplets could detach more easily from the hydrophobic surface. Therefore, the two key factors (vibration and surface properties) play a critical role in the formation of hierarchical structures.

From the perspective of superhydrophobic surface design, forming hierarchical structures directly on the micropillar surfaces is essential, which can be explained in terms of the Cassie–Baxter state [32, 33, 34, 35]. The state of a water droplet on the microstructures can be predicted by the following equation:

$$\cos\theta_{A}=-1+f\left(1+\cos\theta \right)$$

where θ_*A*_ is the apparent contact angle on the microstructured surface, *f* is the fraction of the solid surface area wetted by the liquid, and θ is the intrinsic contact angle on a flat surface. When a hierarchical structure is formed on the existing microstructure due to water vapor condensation, the water droplet is positioned on top of the hierarchical structure. As a result, the *f* value decreases compared to the control, causing the value of *cos*θ_*A*_ to approach ‐1, and consequently, the θ_*A*_ value increases in the hierarchical structures case. Thus, fabricating hierarchical structures on micropillars is crucial for achieving superhydrophobicity.

### 3.3 Wettability

**FIGURE 4:**
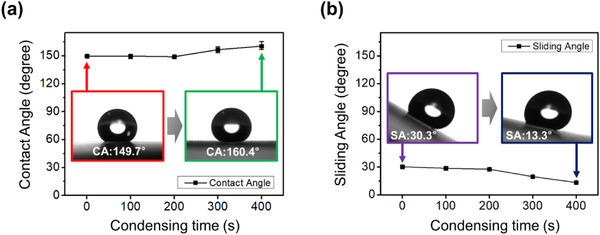
Wettability of the hierarchical structures fabricated via water condensation. (a) Contact angle measurement. (b) Sliding angle measurement.

As with most micro‐hierarchical structures, the fabricated hierarchical structures via water vapor condensation also exhibited superhydrophobic behavior, characterized by a high contact angle, a low sliding angle, and a “pancake” shape during droplet impact. To investigate the characteristics of superhydrophobic surfaces, various wettability tests were implemented. Figure 4 presents the wetting characteristics of the hierarchical structures. The contact angle measurements revealed values of 149.7°, 149.0°, 156.6°, and 160.4° for samples fabricated with condensation times of 100 s, 200 s, 300 s, and 400 s, respectively (Figure 4a; Figure S7a). The control sample (micro‐pillars only) exhibited a contact angle of 149.7°. The 100 s and 200 s samples, which showed negligible hierarchical formation, maintained CAs similar to the control. In contrast, the 300 s and 400 s samples exhibited significantly improved CAs of 156.9° and 160.4°, respectively. This enhancement is attributed to the growth of the average droplet diameter (from 11.2 µm to 19.5 µm) and a ∼72% increase in number density as condensation time progressed from 200 s to 400 s (Table 1). These structural changes effectively reduced the solid‐liquid contact area fraction, thereby stabilizing the water droplet in a Cassie‐Baxter state. Consequently, the 400 s sample showed a 10.7° increase in CA compared to the control, demonstrating that the dual‐level hierarchical structuring significantly enhances superhydrophobicity.

Moreover, sliding angle measurements further confirmed this trend (Figure 4b). The control exhibited a high sliding angle of 30.3°, while the hierarchical samples showed reduced sliding angles of 28.7°, 27.7°, 19.7°, and 13.3° for 100, 200, 300, and 400 s, respectively (Figure S7b). Notably, the sliding angle was greatly improved from 30.3° (control) to 13.3° (400 s sample), showing a decrease of 17°. Quantitatively, the 400 s sample exhibits a high number density of 693 droplets per unit area, with an inter‐droplet distance significantly smaller than those of the other samples. This dense distribution establishes a stable Cassie‐Baxter state by effectively trapping air pockets within the hierarchical roughness, thereby facilitating easy droplet roll‐off (low sliding angle). In addition, this behavior can be interpreted in terms of the pinning–depinning dynamics of the contact line on rough surfaces [9, 36, 37]. When a droplet moves across a microstructured surface, the contact line becomes intermittently pinned at local energy minima, and an energy barrier must be overcome for motion to continue. The formation of hierarchical structures decreases the practical contact area and reduces the work of adhesion between the droplet and the surface, which consequently lowers the energy barrier for depinning. As a result, the stored elastic energy in the deformed droplet is released more efficiently during each depinning event, allowing the droplet to translate more smoothly across the surface. According to the energy balance model, when the surface exhibits a large dynamic receding angle, most of the released energy is dissipated along the surface normal, and only a small portion contributes to lateral motion [9]. However, on the hierarchical superhydrophobic surface, reduced pinning and a lower adhesion energy enable a greater fraction of the interfacial energy to be converted into translational motion, resulting in a smaller sliding angle. Therefore, both the geometric roughness and the reduced surface adhesion synergistically enhance droplet mobility on the hierarchical surface.

**FIGURE 5:**
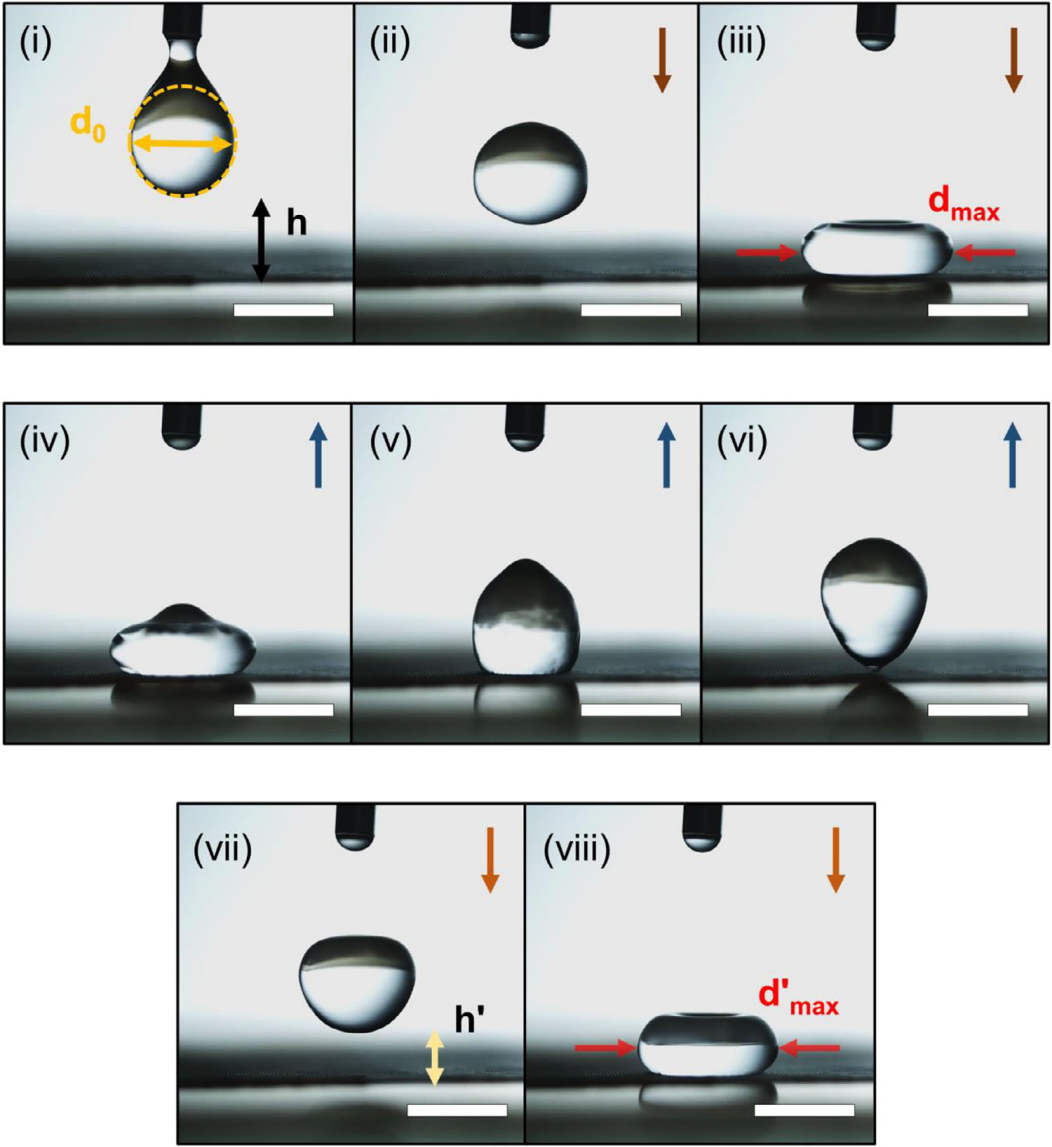
Bouncing behavior of a water droplet on the hierarchical surface. Images were recorded during 100 ms. (i–iii) Initial impact (iv–vi) rebound process, and (vii–viii) second impact. Scale bars are 3 mm.

To further evaluate the water‐repellent properties of the superhydrophobic surface, high‐speed imaging of a droplet impact was performed using a sample fabricated with a 400 s condensation time (Figure 5). Based on the results shown in Figure 4, the droplet impact and rebound dynamics were observed over a period of 100 ms. Figure 5i–iii illustrate the initial droplet impact, Figure 5 iv–vi show the rebound process, and Figure 5 vii,viii capture the second impact. During the initial impact, a droplet with a diameter of 3.3 mm fell from a height of 2.6 mm, spreading into a pancake‐shaped droplet with a diameter of 4.8 mm (Figure 5‐iii). In this experiment, the pancake shape and rebounding of the droplet were observed due to water repellency [38, 39]. As shown in Figure 5‐iv–vi, the droplet rebounded to a height of 1.5 mm before falling again, forming a second pancake with a diameter of 4.5 mm. The pancake morphology of bouncing droplets can be explained using the Weber number (*We*), which can predict the impact droplet quality [39, 40]:

$$\mathrm{We}=\frac{\rho v_{0}^{2}d_{0}}{2\gamma }$$

where *d_0_* is the initial droplet diameter (3.3 mm), *v_0_* is the impact velocity, and *ρ* and *γ* are the density (1000 kg m^−^^3^) and surface energy of water, respectively. From potential energy considerations, the impact velocities at 2.6 mm and 1.5 mm heights were calculated as 0.25 m s^−^^1^ and 0.17 m s^−^^1^, respectively, yielding *We* values of 1.47 and 0.67. These results imply that the pancake shape formed during the first droplet impact was flatter than that formed during the second.

Furthermore, by calculating the coefficient of restitution, it is possible to verify whether the superhydrophobic surface exhibits excellent water repellency. The water droplet bouncing can be explained using the energy conservation [38, 41, 42]. When the water droplet impacts certain surfaces, the potential energy of the water droplet is changed to kinetic energy, and this energy relationship can be expressed as follows,

$$E_{k,0}+E_{s,0}=E_{k,1}+E_{s,1}+E_{\mathrm{dis}}$$

where *E*_k,0_ and *E*_s,0_ are the kinetic/surface energies of the water droplet at the first impact, and *E*_k,1_ and *E*_s,1_ are the kinetic/surface energies of the water droplet at rebound from the surface. *E*_dis_ is an energy dissipation. The key factor for evaluating the superhydrophobic surfaces is *E*_dis_. When a droplet impacts a surface, it spreads out, increasing its contact area with the surface, and during this process, energy loss occurs. Typically, superhydrophobic surfaces exhibit low friction with water droplets, resulting in less energy loss. Therefore, on superhydrophobic surfaces with excellent water‐repellent performance, a higher coefficient of restitution value is obtained. The coefficient of restitution (*e*) was calculated using [43, 44]:

$$\frac{H_{r}}{H}=e^{2}$$

where *H_r_* is the rebound height and *H* is the initial height. From literature, a value of e between 0.6 and 0.9 generally indicates an excellent superhydrophobic surface [43]. For the hierarchical structure in this study, *e* was calculated as 0.76, confirming superior superhydrophobicity.

## 4 Application

**FIGURE 6:**
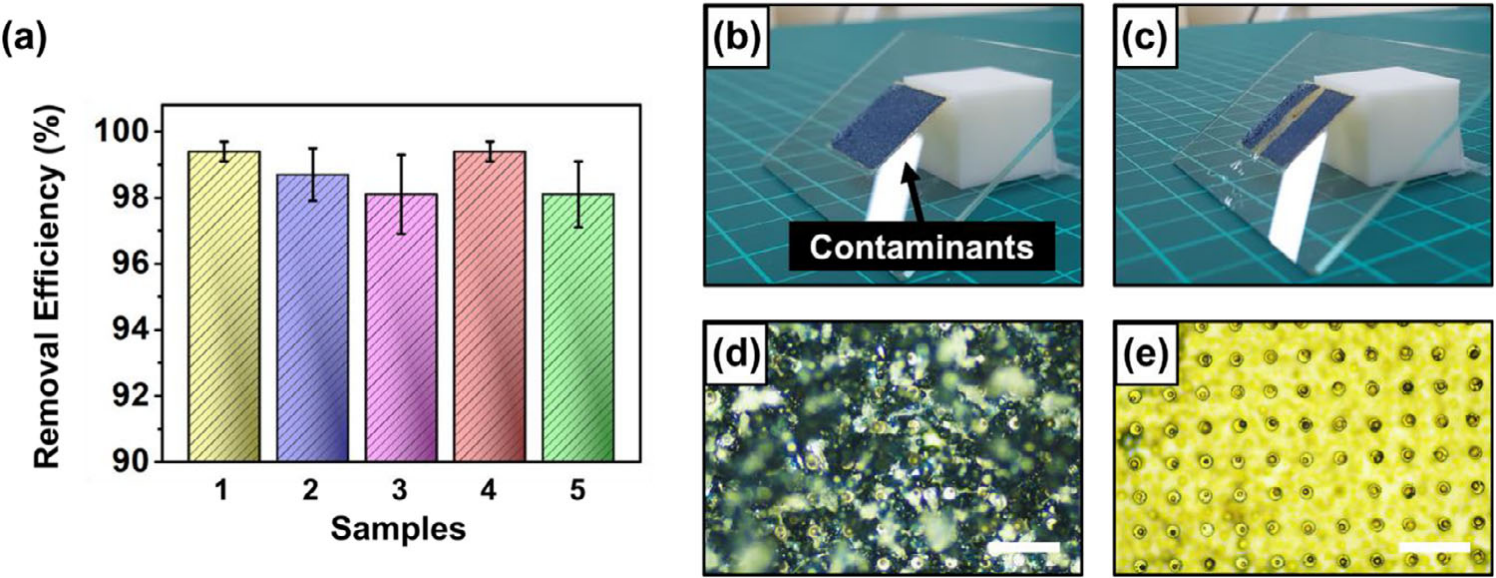
Self‐cleaning test of the hierarchical surface. (a) Quantitative analysis of contaminant removal efficiency. Photographs of (b) the initial state and (c) the cleaned state. Optical microscope images of (d) the initial state and (e) the cleaned state. Scale bars are 200 µm.

The excellent wetting properties of the fabricated superhydrophobic surface were previously validated by the results shown in Figures 4 and 5. One of the most remarkable properties of superhydrophobic surfaces is their self‐cleaning ability [45, 46, 47, 48]. Thus, to further verify the performance of the fabricated surface as a feasible application, a simple self‐cleaning test was conducted (Figure 6). After uniformly spreading contaminants on the sample fabricated with a 400 s condensation time, 0.5 mL of water was dropped onto the surface at a tilt angle of 37° (Figure 6b,c). As shown in Figure 6d,e, the initially contaminated surface was thoroughly cleaned after the test. As shown in Figure 6a, even with a small amount of water, excellent cleaning performance as high as 98.7% was achieved. Through these experiments, the outstanding self‐cleaning property of the superhydrophobic surface was successfully demonstrated, and the hierarchical structure fabrication method via water vapor condensation was comprehensively validated, from fundamental wetting properties to practical applications such as self‐cleaning.

## 5 Conclusion

In this study, a facile fabrication method for superhydrophobic surfaces via water vapor condensation is proposed. To make the dual‐level hierarchical structures, water droplets were induced onto a micropillar array (backbone layer) through the condensation of water vapor, followed by freezing of the droplets. Using PDMS and PUA, the hierarchical structures were successfully replicated. The morphology of the hierarchical structures was controlled by varying the condensation time, and the optimized sample with a condensation time of 400 s was determined. After surface modification using L‐SAM, a superhydrophobic surface was finally achieved.

To investigate the characteristics of the superhydrophobic hierarchical structures, various surface properties related to wettability were examined from both experimental and theoretical perspectives. The behavior of water droplets on the superhydrophobic surface was observed and analyzed based on the Cassie–Baxter state. The contact angle and sliding angle of the 400 s–condensed sample were measured to be approximately 160.4° and 13.3°, respectively.

To further confirm the superhydrophobicity of the fabricated sample, a water‐repellency test was conducted using a high‐speed camera. When a water droplet impacted the surface, the “pancake” deformation—one of the representative phenomena of superhydrophobic surfaces in the water drop impact test—was clearly observed. This behavior was analyzed in terms of energy conservation and the Weber number, and the coefficient of restitution of the droplet was calculated to be 0.76. These results indicate that the sample fabricated via water vapor condensation clearly exhibits the characteristics of a superhydrophobic surface.

Finally, a simple self‐cleaning test was performed as a practical demonstration of the surface functionality. Contaminants on the surface were effectively removed by a 0.5 mL water droplet, achieving a high particle removal efficiency of approximately 98.7%.

In summary, this study presents a facile method for fabricating superhydrophobic surfaces via water vapor condensation and provides comprehensive analyses of their wettability characteristics. This simple fabrication approach is expected to offer valuable insights not only for hierarchical structure fabrication but also for broader research areas related to surface wettability.

## Conflicts of Interest

The authors declare no conflict of interest.

## Supporting information




**Supporting File 1**: smtd70576‐sup‐0001‐SuppMat.docx.

**Supporting File 2**: smtd70576‐sup‐0002‐VideoS1.mp4.

## Data Availability

The data that support the findings of this study are available from the corresponding author upon reasonable request.

## References

[1] M. Yamamoto , N. Nishikawa , H. Mayama , et al., “Theoretical Explanation of the Lotus Effect: Superhydrophobic Property Changes by Removal of Nanostructures From the Surface of a Lotus Leaf,” Langmuir 31 (2015): 7355–7363, 10.1021/acs.langmuir.5b00670. 26075949

[2] M. Zhang , S. Feng , L. Wang , and Y. Zheng , “Lotus Effect in Wetting and Self‐Cleaning,” Biotribology 5 (2016): 31–43.

[3] A. Marmur , “The Lotus Effect: Superhydrophobicity and Metastability,” Langmuir 20 (2004): 3517–3519, 10.1021/la036369u. 15875376

[4] D. Wang , Q. Sun , M. J. Hokkanen , et al., “Design of Robust Superhydrophobic Surfaces,” Nature 582 (2020): 55–59, 10.1038/s41586-020-2331-8. 32494077

[5] J. Jeevahan , M. Chandrasekaran , G. Britto Joseph , R. Durairaj , and G. Mageshwaran , “Superhydrophobic Surfaces: A Review on Fundamentals, Applications, and Challenges,” Journal of Coatings Technology and Research 15 (2018): 231–250, 10.1007/s11998-017-0011-x.

[6] P. Roach , N. J. Shirtcliffe , and M. I. Newton , “Progess in Superhydrophobic Surface Development,” Soft Matter 4 (2008): 224–240, 10.1039/B712575P. 32907233

[7] M. Ma and R. M. Hill , “Superhydrophobic Surfaces,” Current Opinion in Colloid & Interface Science 11 (2006): 193–202, 10.1016/j.cocis.2006.06.002.

[8] B. J. Ryan and K. M. Poduska , “Roughness Effects on Contact Angle Measurements,” American Journal of Physics 76 (2008): 1074–1077, 10.1119/1.2952446.

[9] P. Olin , S. B. Lindström , T. Pettersson , and L. Wagberg , “Water Drop Friction on Superhydrophobic Surfaces,” Langmuir 29 (2013): 9079–9089, 10.1021/la401152b. 23721176

[10] A. Cassie and S. Baxter , “Wettability of Porous Surfaces,” Transactions of the Faraday Society 40 (1944): 546, 10.1039/tf9444000546.

[11] S. H. Lee , B. S. Kang , and M. K. Kwak , “Magneto‐Responsive Actuating Surfaces With Controlled Wettability and Optical Transmittance,” ACS Applied Materials & Interfaces 14 (2022): 14721–14728, 10.1021/acsami.1c24556. 35289610

[12] C. I. Park , H. E. Jeong , S. H. Lee , H. S. Cho , and K. Y. Suh , “Wetting Transition and Optimal Design for Microstructured Surfaces with Hydrophobic and Hydrophilic Materials,” Journal of Colloid and Interface Science 336 (2009): 298–303, 10.1016/j.jcis.2009.04.022. 19426991

[13] Y. Noh , S. Park , and S. Lee , “AI‐Assisted 3D Modeling Strategy for Microstructure‐Based Functional Surfaces Using ChatGPT and Random Forest,” Machines 12 (2024): 930.

[14] D. Ebert and B. Bhushan , “Durable Lotus‐Effect Surfaces with Hierarchical Structure Using Micro‐ and Nanosized Hydrophobic Silica Particles,” Journal of Colloid and Interface Science 368 (2012): 584–591, 10.1016/j.jcis.2011.09.049. 22062688

[15] W. Li and A. Amirfazli , “Hierarchical Structures for Natural Superhydrophobic Surfaces,” Soft Matter 4 (2008): 462–466, 10.1039/B715731B. 32907205

[16] Y. Lee , S. H. Park , K. B. Kim , and J. K. Lee , “Fabrication of Hierarchical Structures on a Polymer Surface to Mimic Natural Superhydrophobic Surfaces,” Advanced Materials 19 (2007): 2330–2335, 10.1002/adma.200700820.

[17] N. Gao , Y. Yan , X. Chen , and D. Mee , “Superhydrophobic Surfaces with Hierarchical Structure,” Materials Letters 65 (2011): 2902–2905, 10.1016/j.matlet.2011.06.088.

[18] J. Xiao , K. Yin , L. Wang , et al., “Femtosecond Laser Atomic–Nano–Micro Fabrication of Biomimetic Perovskite Quantum Dots Films Toward Durable Multicolor Display,” ACS Nano 19 (2025): 23431–23441, 10.1021/acsnano.5c06945. 40536059

[19] B. Zhou , K. Yin , J. Xiao , et al., “Anti‐Frosting Perovskite Quantum Dots Films via Femtosecond Laser Composite Texturing,” Advanced Functional Materials 36 (2025): 14663.

[20] L. Wang , K. Yin , Q. Deng , Q. Huang , J. He , and J. A. Duan , “Wetting Ridge‐Guided Directional Water Self‐Transport,” Advanced Science 9 (2022): 2204891, 10.1002/advs.202204891. 36253156 PMC9731720

[21] K. Yin , D. Chu , X. Dong , C. Wang , J.‐A. Duan , and J. He , “Femtosecond Laser Induced Robust Periodic Nanoripple Structured Mesh for Highly Efficient Oil–Water Separation,” Nanoscale 9 (2017): 14229–14235, 10.1039/C7NR04582D. 28914319

[22] K. J. Regehr , M. Domenech , J. T. Koepsel , et al., “Biological Implications of Polydimethylsiloxane‐Based Microfluidic Cell Culture,” Lab on a Chip 9 (2009): 2132, 10.1039/b903043c. 19606288 PMC2792742

[23] E. E. Hamurcu and B. M. Baysal , “Solubility Parameter of a Poly(dimethylsiloxane) Network,” Journal of Polymer Science Part B: Polymer Physics 32 (1994): 591–594, 10.1002/polb.1994.090320322.

[24] J. N. Lee , C. Park , and G. M. Whitesides , “Solvent Compatibility of Poly(dimethylsiloxane)‐Based Microfluidic Devices,” Analytical Chemistry 75 (2003): 6544–6554, 10.1021/ac0346712. 14640726

[25] S. H. Lee , H. W. Song , H. J. Park , and M. K. Kwak , “Surface Adaptable and Adhesion Controllable Dry Adhesive with Shape Memory Polymer,” Macromolecular Rapid Communications 43 (2022): 2200012, 10.1002/marc.202200012. 35132723

[26] W. K. Lin , S. Liu , S. Lee , et al., “High Q‐Factor Polymer Microring Resonators Realized by Versatile Damascene Soft Nanoimprinting Lithography,” Advanced Functional Materials 34 (2024): 2312229, 10.1002/adfm.202312229. 39022395 PMC11251712

[27] S. Zhang , L. Zhao , M. Yu , et al., “Measurement Methods for Droplet Adhesion Characteristics and Micrometer‐Scale Quantification of Contact Angle on Superhydrophobic Surfaces: Challenges and Opportunities,” Langmuir 40 (2024): 9873–9891, 10.1021/acs.langmuir.3c03967. 38695884

[28] H. Fox and W. Zisman , “The Spreading of Liquids on Low Energy Surfaces. I. Polytetrafluoroethylene,” Journal of Colloid Science 5 (1950): 514–531, 10.1016/0095-8522(50)90044-4.

[29] H. Murase , K. Nanishi , H. Kogure , T. Fujibayashi , K. Tamura , and N. Haruta , “Interactions Between Heterogeneous Surfaces of Polymers and Water,” Journal of Applied Polymer Science 54 (1994): 2051–2062, 10.1002/app.1994.070541307.

[30] L. Gao and T. J. McCarthy , “Teflon is Hydrophilic. Comments on Definitions of Hydrophobic, Shear versus Tensile Hydrophobicity, and Wettability Characterization,” Langmuir 24 (2008): 9183–9188, 10.1021/la8014578. 18672918

[31] C. H. Kung , P. K. Sow , B. Zahiri , and W. Mérida , “Assessment and Interpretation of Surface Wettability Based on Sessile Droplet Contact Angle Measurement: Challenges and Opportunities,” Advanced Materials Interfaces 6 (2019): 1900839, 10.1002/admi.201900839.

[32] K. L. Wilke , Y. Song , Z. Lu , and E. N. Wang , “Enhanced Laplace Pressures for Functional Surfaces: Wicking, Switchability, and Selectivity,” Advanced Materials Interfaces 10 (2023): 2201967, 10.1002/admi.202201967.

[33] S. H. Lee , J. H. Lee , C. W. Park , et al., “Continuous Fabrication of Bio‐Inspired Water Collecting Surface via Roll‐Type Photolithography,” International Journal of Precision Engineering and Manufacturing‐Green Technology 1 (2014): 119–124, 10.1007/s40684-014-0016-1.

[34] K. Lin , D. Zang , X. Geng , and Z. Chen , “Revisiting the Effect of Hierarchical Structure on the Superhydrophobicity,” The European Physical Journal E 39 (2016): 15, 10.1140/epje/i2016-16015-8. 26920518

[35] Y. Xue , S. Chu , P. Lv , and H. Duan , “Importance of Hierarchical Structures in Wetting Stability on Submersed Superhydrophobic Surfaces,” Langmuir 28 (2012): 9440–9450, 10.1021/la300331e. 22642584

[36] P. Kumar and D. J. Harvie , “Energy Dissipation During Wenzel Wetting via Roughness Scale Interface Dynamics,” Langmuir 40 (2024): 16190–16207, 10.1021/acs.langmuir.4c01292. 39049496

[37] B. M. Mognetti and J. Yeomans , “Modeling Receding Contact Lines on Superhydrophobic Surfaces,” Langmuir 26 (2010): 18162–18168, 10.1021/la103539m. 21067143

[38] Y. Liu , L. Moevius , X. Xu , T. Qian , J. M. Yeomans , and Z. Wang , “Pancake Bouncing on Superhydrophobic Surfaces,” Nature Physics 10 (2014): 515–519, 10.1038/nphys2980. 28553363 PMC5444522

[39] S. H. Lee , B. S. Kang , and M. K. Kwak , “Facile Design and Realization of Extremely Water‐Repellent Surface by Mimicking the Greta oto's Wings,” International Journal of Mechanical Sciences 222 (2022): 107218, 10.1016/j.ijmecsci.2022.107218.

[40] M. Može , S. Jereb , R. Lovšin , J. Berce , M. Zupančič , and I. Golobič , Data in brief 61 (2025): 111697. 40521143 10.1016/j.dib.2025.111697 PMC12163178

[41] S.‐H. Lee , M. Seong , M. K. Kwak , et al., “Tunable Multimodal Drop Bouncing Dynamics and Anti‐Icing Performance of a Magnetically Responsive Hair Array,” ACS Nano 12 (2018): 10693–10702, 10.1021/acsnano.8b05109. 30248255

[42] Y. Liu , G. Whyman , E. Bormashenko , C. Hao , and Z. Wang , “Controlling Drop Bouncing Using Surfaces with Gradient Features,” Applied Physics Letters 107 (2015): 107.

[43] P. K. Sharma and H. N. Dixit , “Energetics of a Bouncing Drop: Coefficient of Restitution, Bubble Entrapment, and Escape,” Physics of Fluids 32 (2020): 112107.

[44] D. Richard , C. Clanet , and D. Quéré , “Contact Time of a Bouncing Drop,” Nature 417 (2002): 811, 10.1038/417811a. 12075341

[45] X. Yang , P. Wang , X. Wu , et al., “A Superhydrophobic Self‐Cleaning Flexible Hydrogel for Solar Thermoelectric Power Generation,” Energy & Fuels 38 (2024): 7887–7898, 10.1021/acs.energyfuels.4c00411.

[46] Z. Yin , T. Zhou , Z. Li , et al., “A Photocatalytic and Superhydrophobic Self‐Cleaning Nanocellulose‐Based Membrane Based on Cu‐MOF for Highly Efficient Oil/Water Separation, the Removal of Dyes and Anti‐Biofouling Towards Sewage Remediation,” International Journal of Biological Macromolecules 322 (2025): 146499, 10.1016/j.ijbiomac.2025.146499. 40754093

[47] S. Rahal , M. D. Choudhury , S. K. Das , D. Samanta , and P. K. Agnihotri , “Designing Lotus‐Like Superhydrophobic Self‐Cleaning Surface Using Carbon Nanotubes,” Physics of Fluids 36 (2024): 107119.

[48] S. P. Dalawai , M. A. S. Aly , S. S. Latthe , et al., “Recent Advances in Durability of Superhydrophobic Self‐Cleaning Technology: A Critical Review,” Progress in Organic Coatings 138 (2020): 105381, 10.1016/j.porgcoat.2019.105381.

