# Supplementary material for: Facile Fabrication of Superhydrophobic Surfaces with Hierarchical Structures via Water Vapor Condensation
Source: Small Methods. 2026 Mar 1;10(6):e02305. doi: 10.1002/smtd.202502305 (PMC13010237; doi:10.1002/smtd.202502305)
Supplement: Supplementary file 1 — Supporting File 1: smtd70576‐sup‐0001‐SuppMat.docx. [file SMTD-10-e02305-s001.docx]

Supporting Information

Facile Fabrication of Superhydrophobic Surfaces with Hierarchical Structures via Water Vapor Condensation

Jeonghan Kang, Seung Yoon Nam, Sungho Lee*

**Contents**

Fig. S1 SEM images of micropillar array (a) Top view, (b) Tilted-view. Scale bar: 200 μm.

**Fig. S2** SEM images showing the morphological evolution of the hierarchical structures with different condensation times (top view) (a) 200 s, (b) 300 s, and (c) 400 s. Scale bar: 20 μm.

**Fig. S3** SEM images showing the morphological evolution of the hierarchical structures with different condensation times (tilted view): (a) 200 s, (b) 300 s, and (c) 400 s. Scale bars: 200 μm (white), 30 μm (red), and 20 μm (black).

**Fig. S4** Measurement results for the hierarchical structure depth on micro-pillars: (a) average depths of 4.5, 12.5, and 20.5 μm were obtained for the 200, 300, and 400 s samples, respectively. SEM images of the hierarchical structures at (b) 200 s, (c) 300 s, and (d) 400 s. Scale bars: 100 μm (red) and 20 μm (white).

**Fig. S5** Hierarchical structures fabricated on different surfaces (condensation time: 400 s). (a) Pristine PUA surface. (b) L-SAM-treated PUA surface. (i) Contact angle of a water droplet, (ii) optical microscope image. Scale bar: 100 μm.

**Fig. S6** Microscopy images demonstrating the formation of hierarchical structures across the entire surface. To represent the large-area uniformity, low-magnification images were acquired at three different locations within a single sample. (a) 200 s, (b) 300 s, and (c) 400 s. Scale bar: 200 μm.

**Fig. S7** Wettability test results with respect to the condensation time. Measurement results of (a) contact angle and (b) sliding angle. (i)–(v) represent samples with different condensation times: 100 s, 200 s, 300 s, and 400 s.

**Fig. S8** Durability test of the superhydrophobic surfaces: Contact angles measured as-fabricated and after 150 days.

**Movie caption**


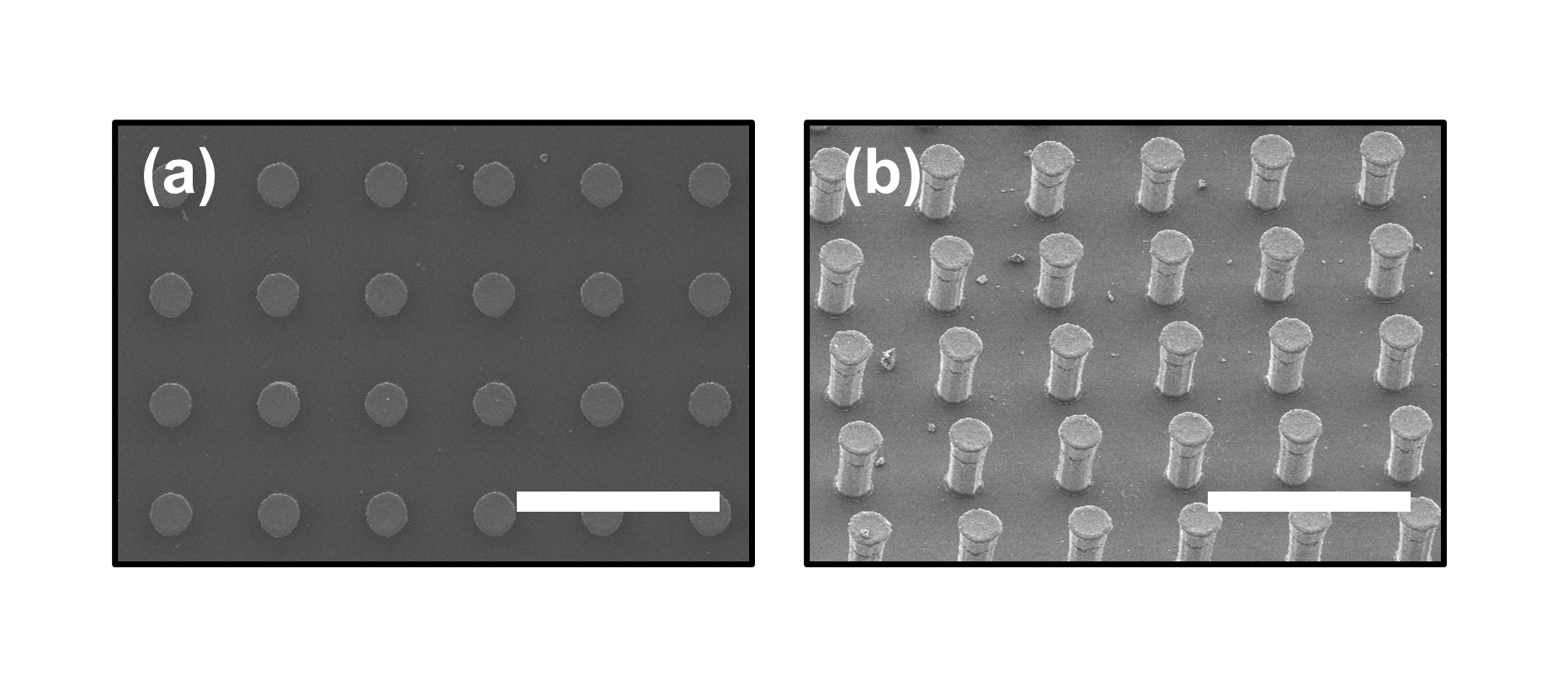


Fig. S1 SEM images of micropillar array (a) Top view, (b) Tilted-view. Scale bar: 200 μm.


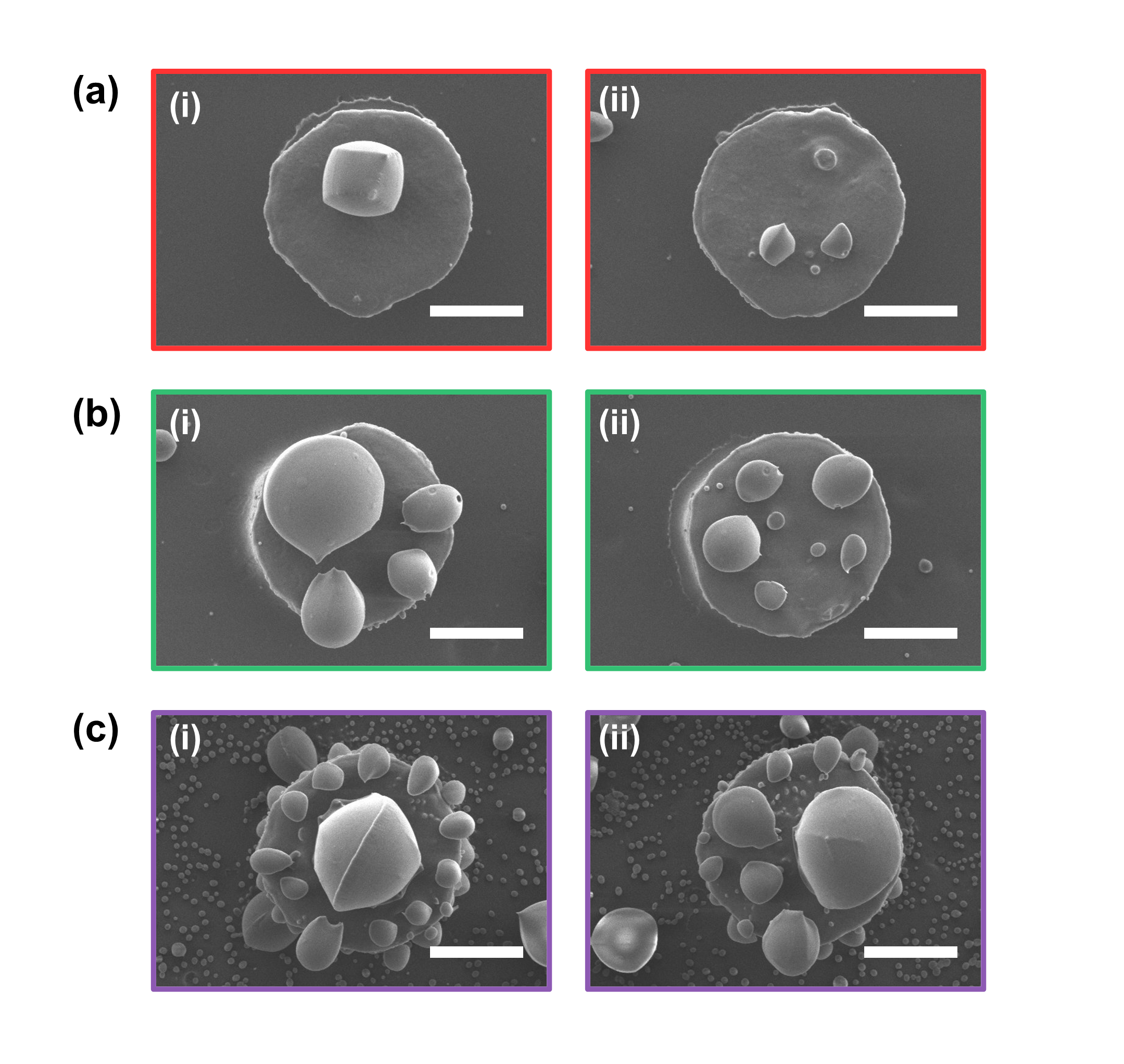


**Fig. S2** SEM images showing the morphological evolution of the hierarchical structures with different condensation times (top view) (a) 200 s, (b) 300 s, and (c) 400 s. Scale bar: 20 μm.


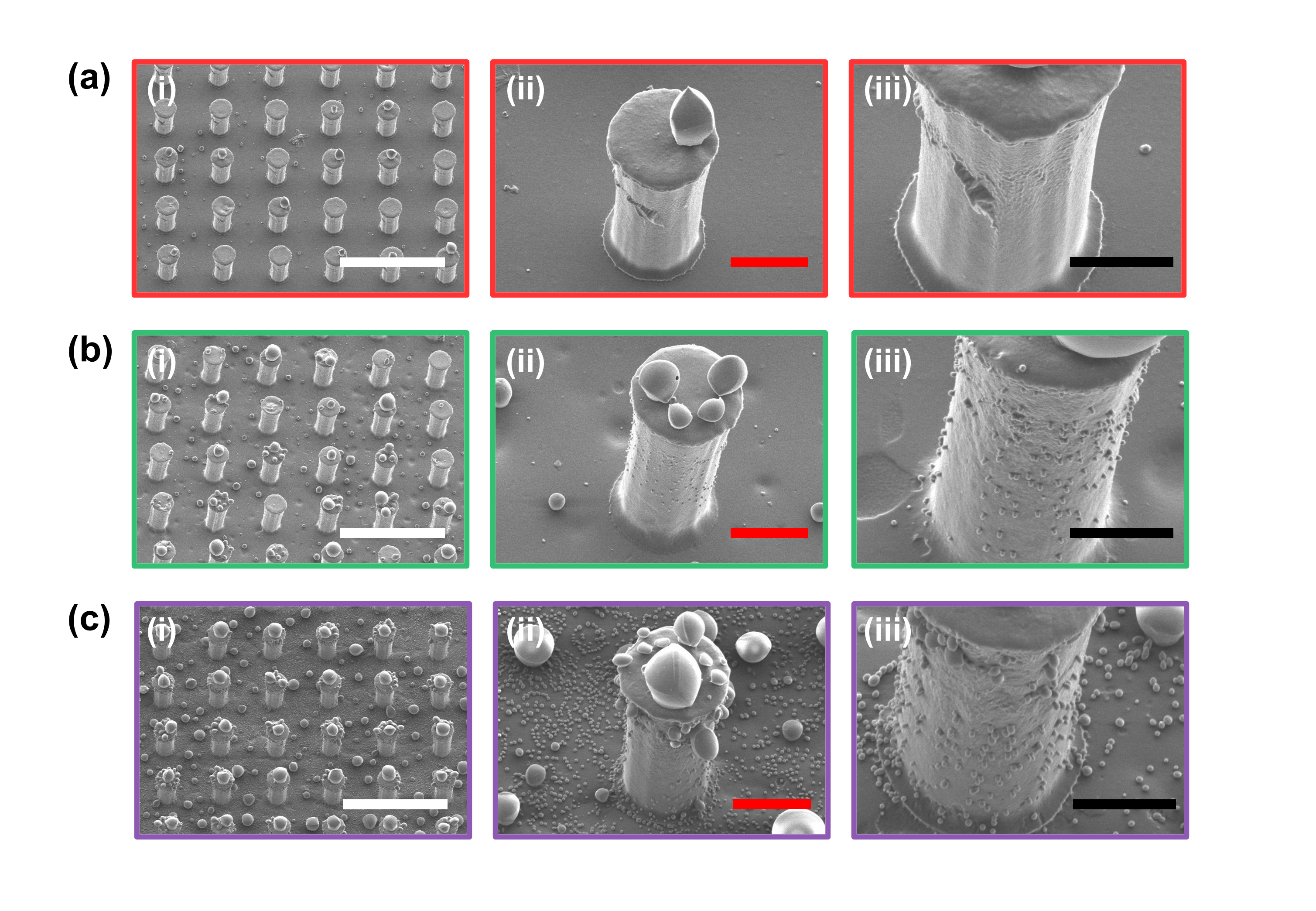


**Fig. S3** SEM images showing the morphological evolution of the hierarchical structures with different condensation times (tilted view): (a) 200 s, (b) 300 s, and (c) 400 s. Scale bars: 200 μm (white), 30 μm (red), and 20 μm (black).


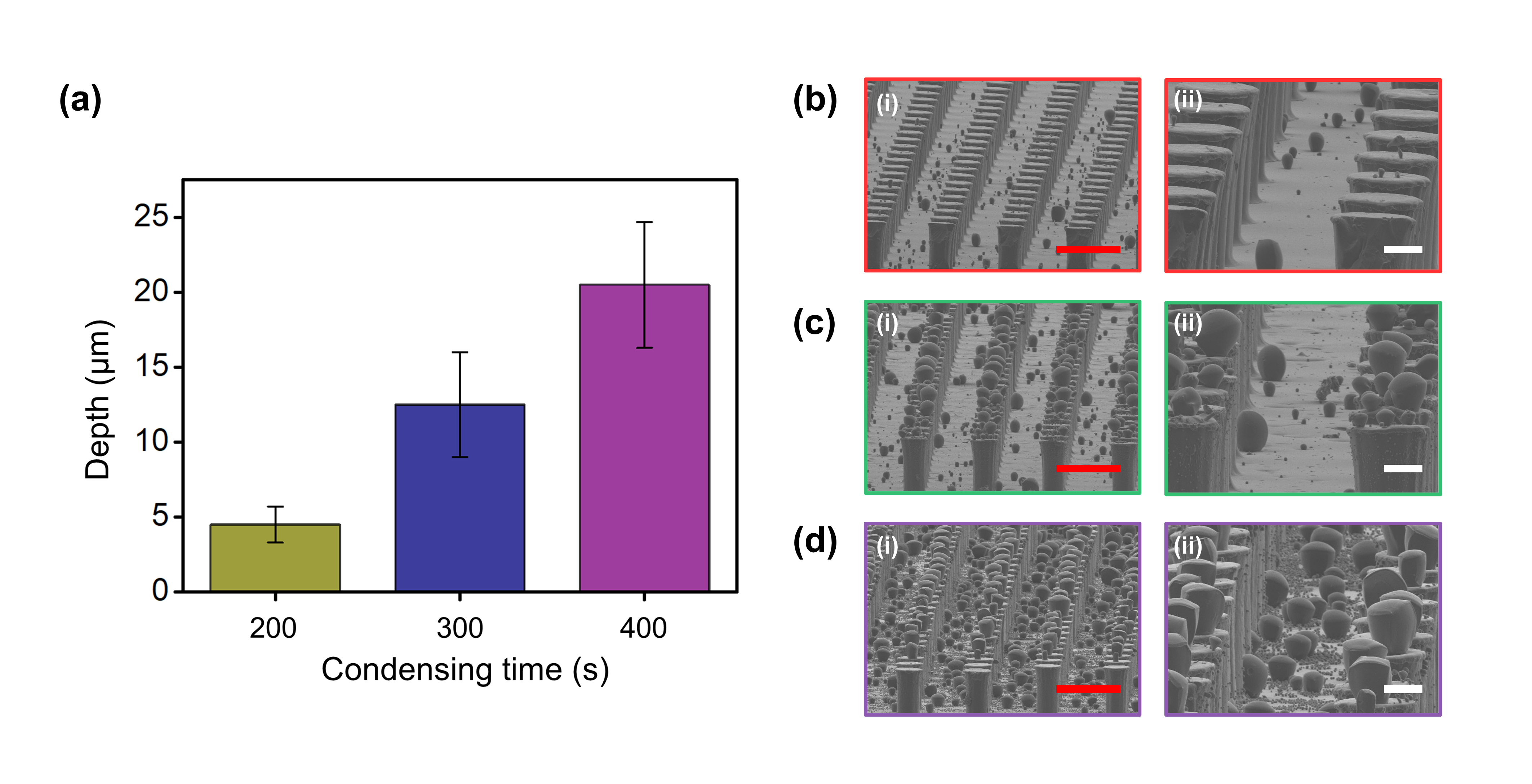


**Fig. S4** Measurement results for the hierarchical structure depth on micro-pillars: (a) average depths of 4.5, 12.5, and 20.5 μm were obtained for the 200, 300, and 400 s samples, respectively. SEM images of the hierarchical structures at (b) 200 s, (c) 300 s, and (d) 400 s. Scale bars: 100 μm (red) and 20 μm (white).


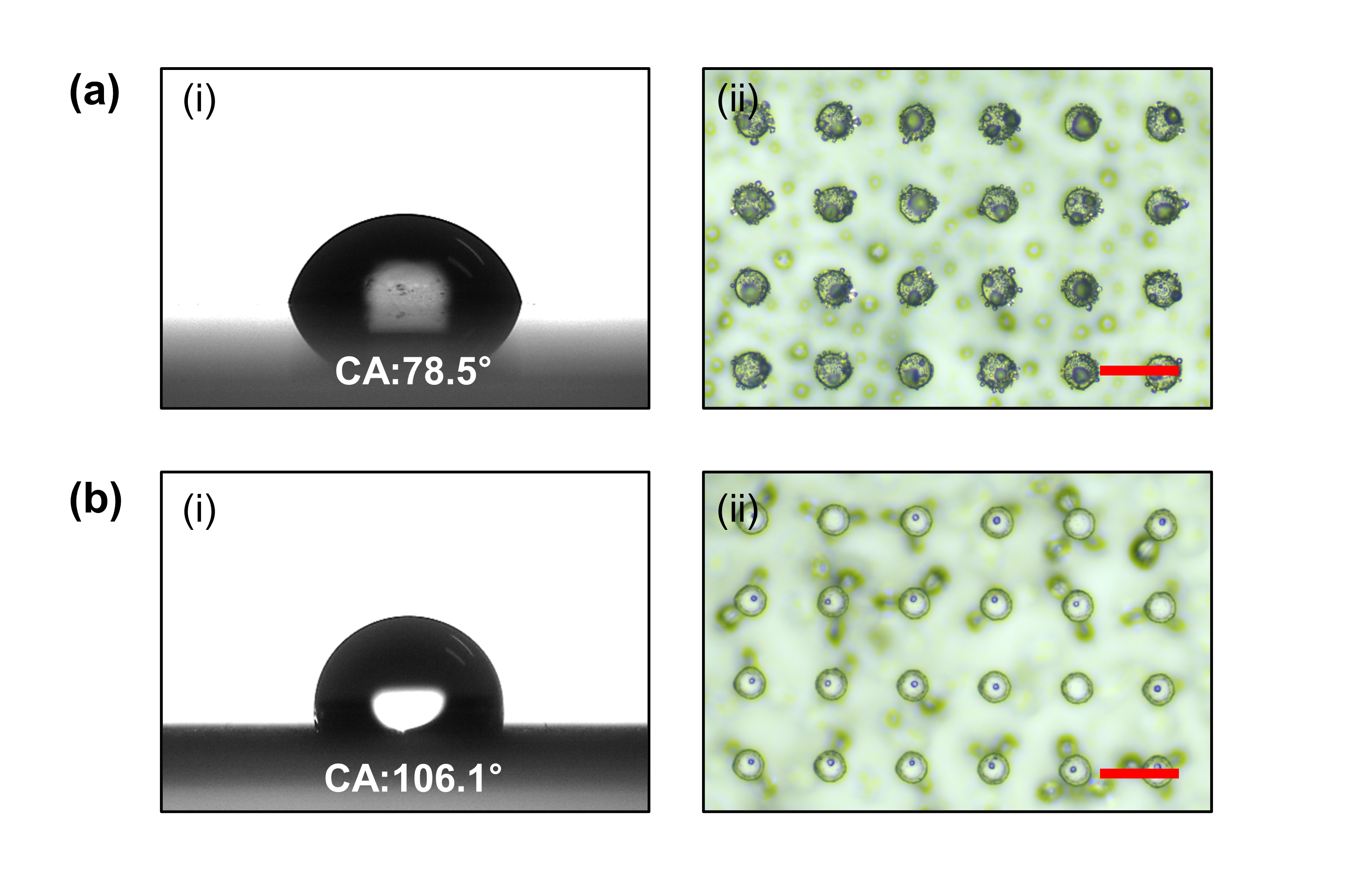


**Fig. S5** Hierarchical structures fabricated on different surfaces (condensation time: 400 s). (a) Pristine PUA surface. (b) L-SAM-treated PUA surface. (i) Contact angle of a water droplet, (ii) optical microscope image. Scale bar: 100 μm.


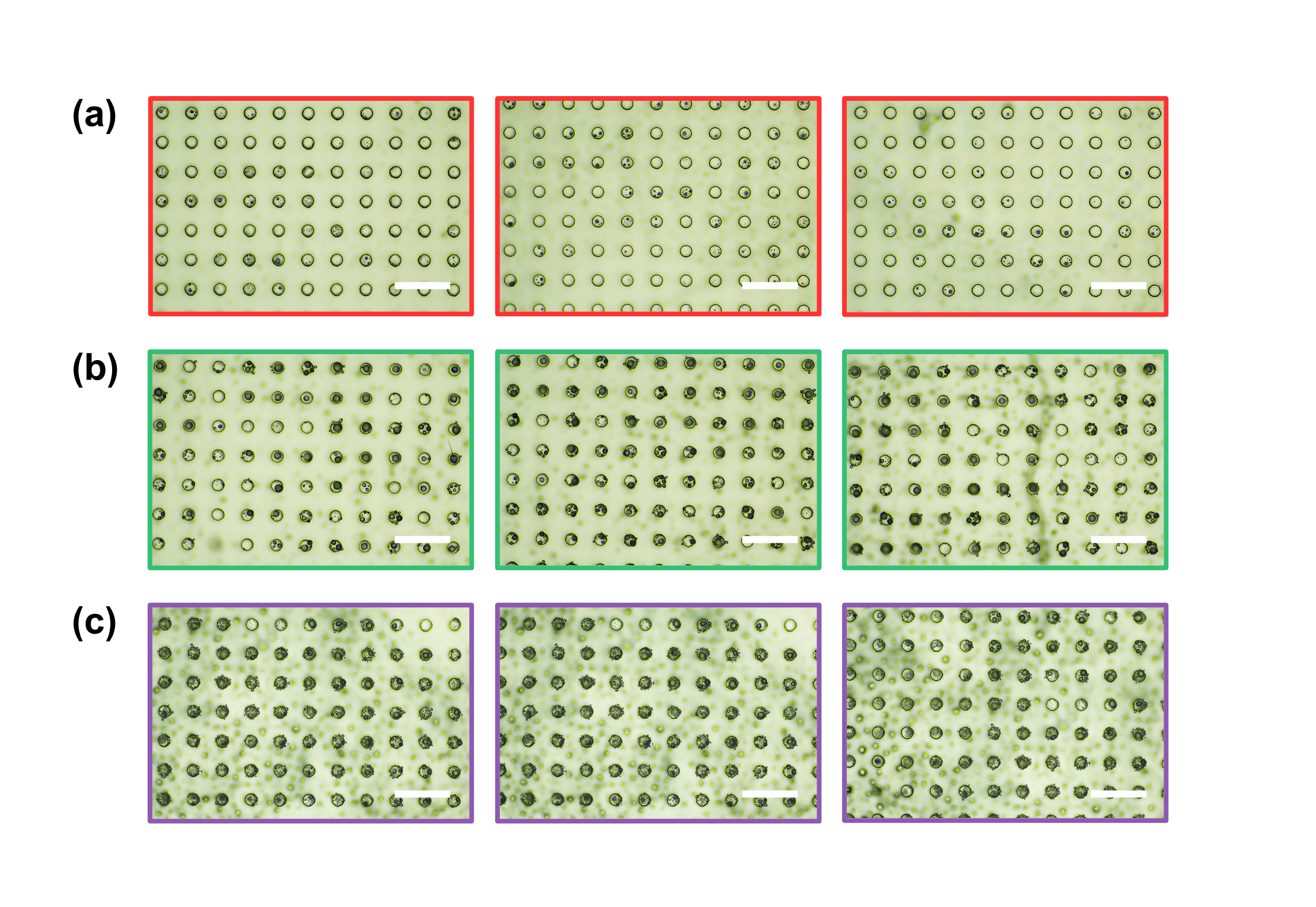
**Fig. S6** Microscopy images demonstrating the formation of hierarchical structures across the entire surface. To represent the large-area uniformity, low-magnification images were acquired at three different locations within a single sample. (a) 200 s, (b) 300 s, and (c) 400 s. Scale bar: 200 μm.


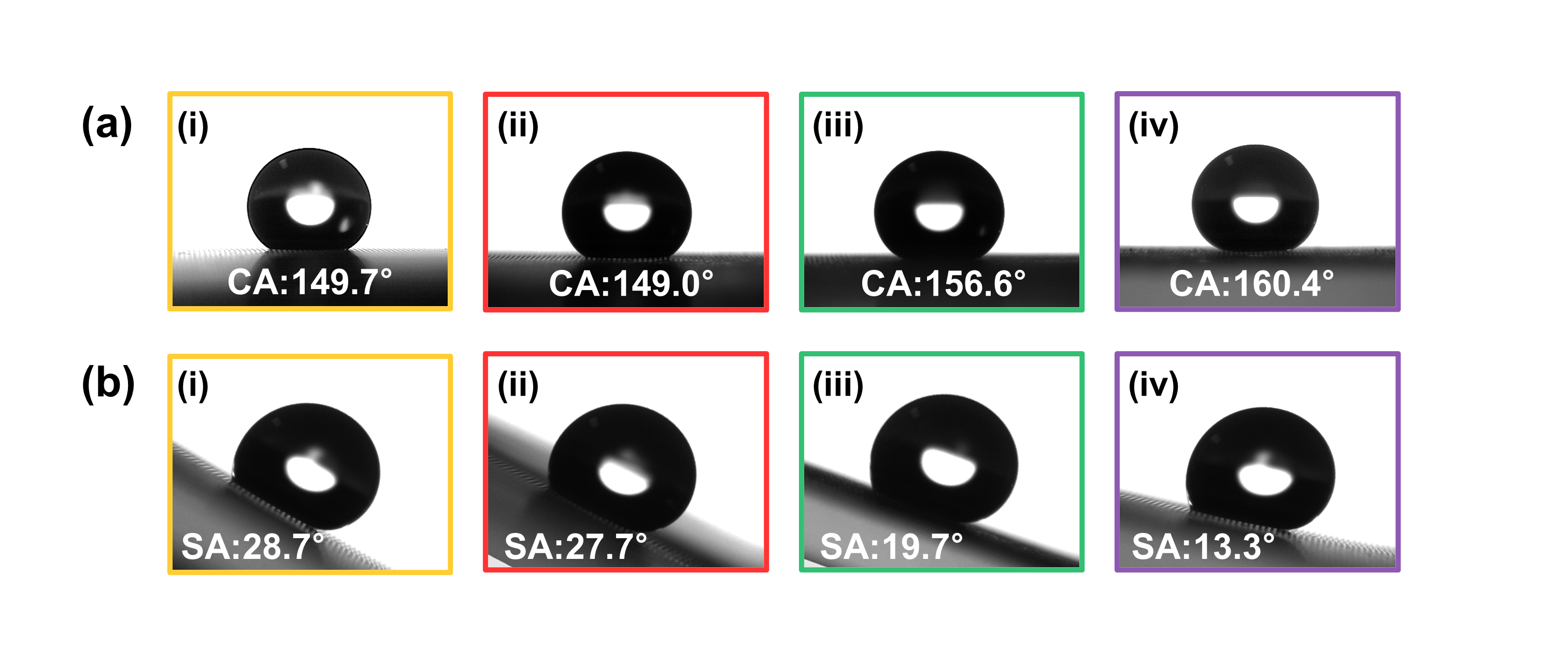


**Fig. S7** Wettability test results with respect to the condensation time. Measurement results of (a) contact angle and (b) sliding angle. (i)–(v) represent samples with different condensation times: 100 s, 200 s, 300 s, and 400 s.


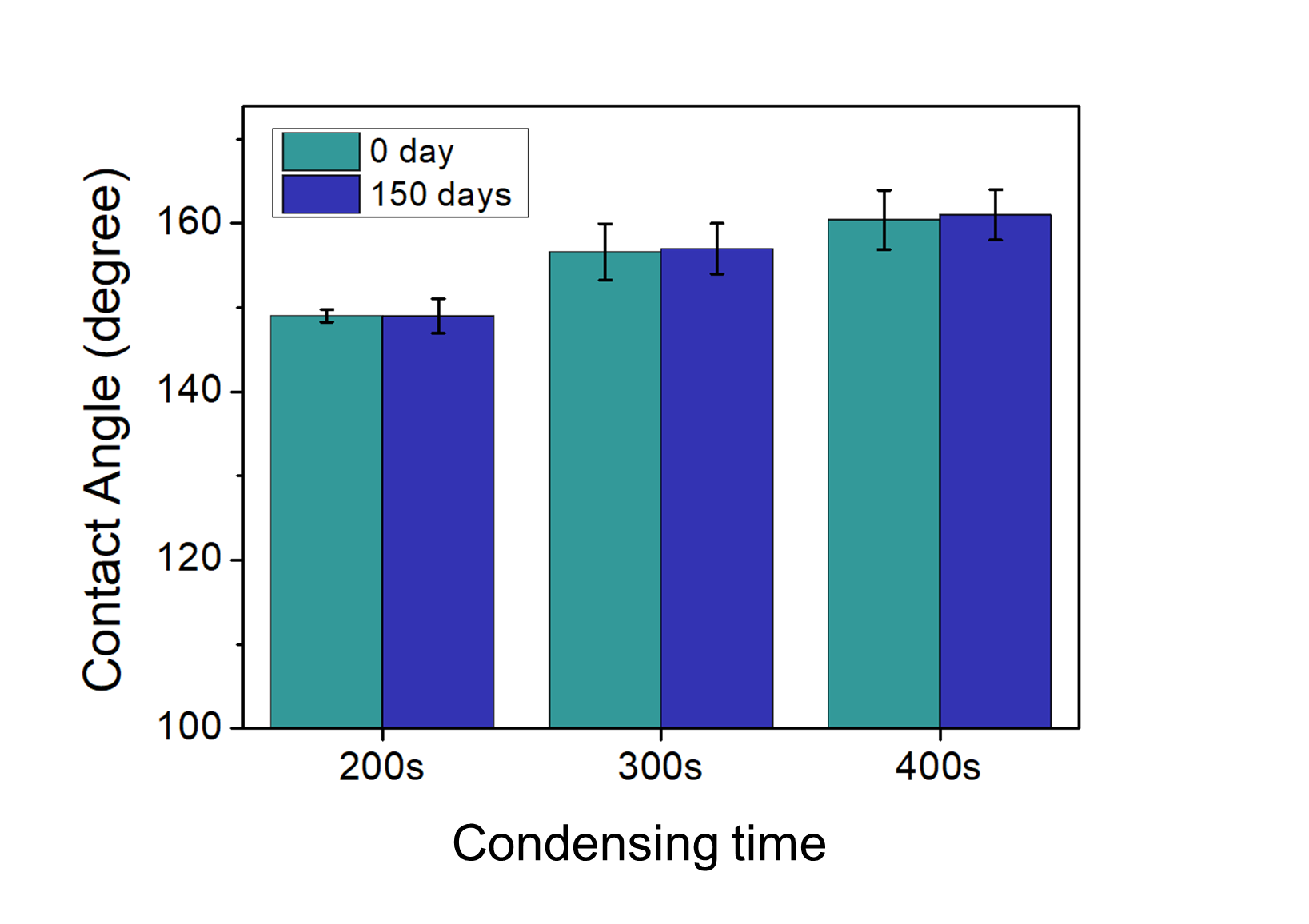


**Fig. S8** Durability test of the superhydrophobic surfaces: Contact angles measured as-fabricated and after 150 days.

**Movie caption**

**• Movie S1**: The “pancake” shape of a water droplet on the hierarchical structures shown in Fig. 5. As observed in Movie S1, the droplet exhibited a distinct pancake shape and rebounded clearly due to the excellent water repellency of the hierarchical structures.
